# Risk factors associated with malaria infection identified through reactive case detection in Zanzibar, 2012–2019

**DOI:** 10.1186/s12936-021-04025-1

**Published:** 2021-12-24

**Authors:** Humphrey R. Mkali, Erik J. Reaves, Shabbir M. Lalji, Abdul-Wahid Al-mafazy, Joseph J. Joseph, Abdullah S. Ali, Faiza B. Abbas, Mohamed H. Ali, Wahida S. Hassan, Chonge Kitojo, Naomi Serbantez, Bilali I. Kabula, Ssanyu S. Nyinondi, Donal Bisanzio, Mike McKay, Erin Eckert, Richard Reithinger, Jeremiah M. Ngondi

**Affiliations:** 1 RTI International, Dar es Salaam, United Republic of Tanzania; 2 U.S. President’s Malaria Initiative, U.S. Centers for Disease Control and Prevention, Dar es Salaam, United Republic of Tanzania; 3 RTI International, Washington DC, USA; 4 Zanzibar Malaria Elimination Programme, Ministry of Health, Zanzibar, United Republic of Tanzania; 5 U.S. President’s Malaria Initiative, United States Agency for International Development, Dar es Salaam, United Republic of Tanzania

**Keywords:** Malaria, Travel, Vector control, Elimination, Zanzibar

## Abstract

**Background:**

Over the past two decades, Zanzibar substantially reduced malaria burden. As malaria decreases, sustainable improvements in control interventions may increasingly depend on accurate knowledge of malaria risk factors to further target interventions. This study aimed to investigate the risk factors associated with malaria infection in Zanzibar.

**Methods:**

Surveillance data from Zanzibar’s Malaria Case Notification system from August 2012 and December 2019 were analyzed. This system collects data on malaria cases passively detected and reported by all health facilities (index cases), and household-based reactive case detection (RCD) activities linked to those primary cases. All members of households of the index cases were screened for malaria using a malaria rapid diagnostic test (RDT). Individuals with a positive RDT were treated with artemisinin-based combination therapy. Univariate and multivariate logistic regression analyses were done to investigate the association between RDT positivity among the household members and explanatory factors with adjustment for seasonality and clustering at *Shehia* level.

**Results:**

A total of 30,647 cases were reported of whom household RCD was completed for 21,443 (63%) index case households and 85,318 household members tested for malaria. The findings show that younger age (*p-value for trend [Ptrend] < 0.001*), history of fever in the last 2 weeks (odds ratio [OR] = 35.7; 95% CI 32.3–39.5), travel outside Zanzibar in the last 30 days (OR = 2.5; 95% CI 2.3–2.8) and living in Unguja (OR = 1.2; 95% CI 1.0–1.5) were independently associated with increased odds of RDT positivity. In contrast, male gender (OR=0.8; 95% CI 0.7–0.9), sleeping under an LLIN the previous night (OR = 0.9; 95% CI 0.7–0.9), having higher household net access (*Ptrend < 0.001*), and living in a household that received IRS in the last 12 months (OR = 0.8; 95% CI 0.7–0.9) were independently associated with reduced odds of RDT positivity. A significant effect modification of combining IRS and LLIN was also noted (OR = 0.7; 95% CI 0.6–0.8).

**Conclusions:**

The findings suggest that vector control remains an important malaria prevention intervention: they underscore the need to maintain universal access to LLINs, the persistent promotion of LLIN use, and application of IRS. Additionally, enhanced behavioural change and preventive strategies targeting children aged 5–14 years and travellers are needed.

## Background

Over the past two decades, Zanzibar substantially reduced malaria burden, with parasite prevalence decreasing from more than 30% in 2005 to 0.2% in 2017 [1]. These gains followed the introduction and rapid scale-up of malaria rapid diagnostic tests (RDTs), artemisinin-based combination therapy (ACT), long-lasting insecticidal nets (LLINs) and indoor residual spraying (IRS) of households with insecticide [2, 3]. The application of malaria control interventions in Zanzibar was aimed at achieving universal coverage and a recent impact evaluation comparing malaria incidence before and after inception of interventions suggested that this approach resulted in rapid declines of malaria [4]. Building on these achievements, Zanzibar is now pursuing malaria elimination by 2023 by maintaining high population-level coverage of malaria interventions, as well as by reinforcing malaria surveillance to actively investigate and classify 100% of confirmed malaria cases [5].

Numerous studies in Africa have examined the association of various demographic and behavioural factors with malaria outcomes. These studies have found that adolescent age, male gender, not sleeping under an LLIN the previous night, and the presence of fever in the 2 weeks preceding the study were positively associated with malaria infection [6, 7]. Similarly, several studies have shown LLIN ownership and use, and/or the application of IRS to be associated with a significant protective effect against malaria infection [6–9]. A recent literature review and meta-analysis of 22 studies investigating travel as a risk factor for malaria infection in sub-Saharan Africa showed that travel—within as well as to and from another country—was an important risk factor in many settings [10].

As malaria decreases, sustainable improvements in prevention and control interventions may increasingly depend on accurate knowledge of malaria risk factors. For Zanzibar to accelerate towards malaria elimination, it is crucial to obtain detailed knowledge of risk factors associated with malaria infection. Such knowledge will help to confirm whether currently implemented preventive and control measures are effective, as well as indicate programmatic areas where additional efforts should be targeted or reinforced.

## Methods

### Study setting and data sources

**Fig. 1:**
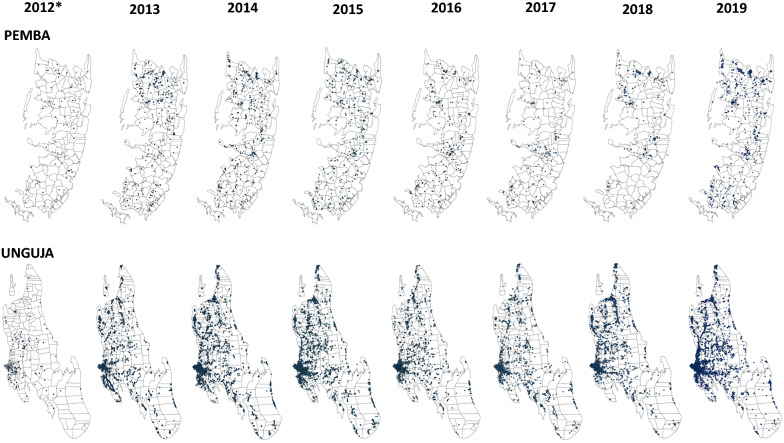
Distribution of location of index case households by Island and year, 2012–2019. *August to December 2012

The archipelago of Zanzibar is located between longitudes 39.19793 and latitudes –6.16394, 25–50 km off the east coast of the Tanzania mainland in the Indian Ocean. There are two main islands—Pemba and Unguja—which cover a total land area of 2461 km^2^ and have an estimated population of 1,303,569 people [11]. There are two main rainfall seasons, a primary (March–May) and a secondary (November–December) season; rainfall is at its lowest in July. Both rainfall seasons are followed by peak malaria transmission seasons, with the highest malaria case count observed in the March–May rainfall season. A cross-sectional analysis of routine data collected by Zanzibar’s Malaria Case Notification (MCN) system between August 2012 and December 2019 was conducted. The MCN system was established in August 2012 with the goal to collect data from all passively detected malaria cases (which we define as index cases); it started in 52 public health facilities and has since progressively scaled-up to 189 public and 124 private health facilities. Case-based follow-up and reactive case detection (RCD) activities, including household-based screening and treatment (HSaT), are linked to index cases through the Coconut surveillance platform (https://coconutsurveillance.org/). Figure 1 (map) shows the location of the households of individuals included in the analysis between 2012 and 2019.

### Data collection

The MCN system collects and reports details of individual malaria cases passively diagnosed by microscopy or RDT at health facilities in real-time using mobile phones and Android tablets to a central database. Once the Council Malaria Surveillance Officer (CMSO) is notified by the health facility worker, surveillance data are collected using an electronic, standardized questionnaire completed by CMSO during routine reactive case follow-up and detection at households of index cases. Household members are revisited if not found at their households during the first household visit. The goal of RCD is to test all household members of the index cases for malaria infection, regardless of presence or absence of symptoms, using RDTs. In most cases and years, the SD Bioline® HRP2/pLDH RDT from Standard Diagnostics, Giheung-ku, Republic of Korea, was used, others used are First Response® Malaria Antigen pLDH/HRP2 Combo card test from Premier Medical Corporation Private Limited, Gujarat, India and Paracheck® Pf—Rapid Test for *P. falciparum* Malaria Device (Ver. 3) from Orchid Biomedical Systems, Goa, India); members with a positive RDT result are treated with ACT following the national malaria diagnosis and treatment guidelines [12]. The specific variables collected are individual factors (i.e., age, gender, self-reported history of travel in the last 30 days, self-reported history of fever in the last 2 weeks, RDT positivity, and LLIN use the previous night); household factors (i.e., number of people residing in the household, number of household LLINs, and IRS application in the last 12 months); and geographical factors (i.e., household geolocation and weekly rainfall). During the study period, rainfall data were obtained from 11 meteorological stations managed by Zanzibar’s Tanzania Meteorological Agency, with rainfall data measured in millimetres (mm) and recorded daily.

### Inclusion and exclusion criteria

**Fig. 2:**
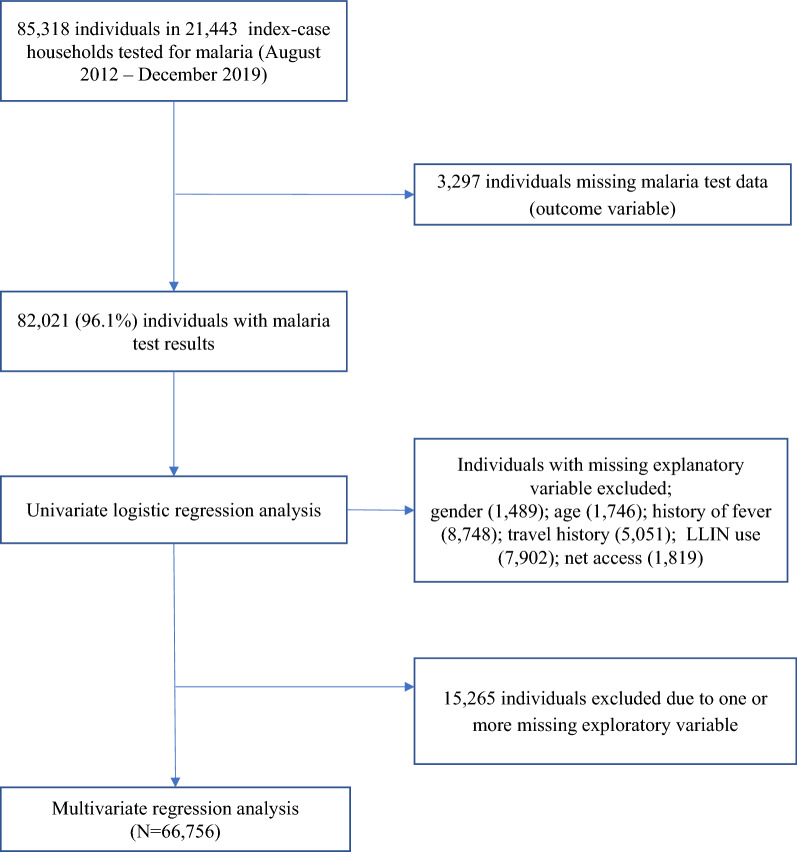
Study individuals included in the analysis from Zanzibar’s Malaria Case Notification and Reactive Case Detection systems, 2012–2019

Inclusion criteria included the index case’s household members identified during routine RCD efforts (i.e., through follow-up of index cases) and tested for malaria infection with an RDT. Individuals were excluded from the analysis if they had missing data for any of the socio-demographic and clinical variables (i.e., individual factors) collected during the RCD efforts. Figure 2 shows the breakdown of included and excluded individuals in the analysis.

### Data analysis

Data analysis was performed using STATA (Version 16, College Station, TX, USA). Net access was defined as the proportion of population with access to LLIN within the household, where an intermediate variable “potential LLIN users” was generated by multiplying the reported number of LLINs in each household by a factor of 2 and divided by the number of household members [13], and a cut-off of ≥ 80% was applied to define universal coverage. Daily rainfall data were aggregated into weekly district-level rainfall data, which was transformed into a continuous variable in 10 mm increments [9]. To account for the lag time between rainfall and malaria cases, we applied the rainfall data for the previous 9 weeks to the current week [14].

Univariate and multivariable logistic regression analyses were conducted to assess the association of RDT positivity and explanatory factors (i.e., age, gender, self-reported fever, net access, travel history, household characteristics, zone, and weekly rainfall) accounting for clustering of cases at *Shehia* (smallest administrative unit) level. The month of household reactive case investigation was included in the multivariable logistic regression to account for variability arising from malaria seasonality. A backward stepwise multiple logistic regression was used to identify independent risk factors for RDT positivity while controlling for potential confounders; the interaction between IRS and LLIN use was also assessed. Lastly, multicollinearity of the model was assessed using variance inflation factor (VIF) for the independent variables, with a VIF less than 5 indicating the model is not biased by multicollinearity of its independent variables.

## Results

### Characteristics of study population

Table 1 summarizes the number of index cases reported, case investigation, household follow-up and household members tested while Fig. 2 shows the number of individuals included/excluded in the analysis. Between August 2012 and December 2019, 30,647 index malaria cases were reported by health facilities through the MCN system; overall, an index case investigation and household RCD was completed for 21,443 (63%) index case households (Table 1). In these 21,443 index case households, 85,318 individuals were investigated, of whom varying numbers were excluded from the analyses due to missing malaria test, socio-demographic and clinical data (Fig. 2). Among the 82,021 individuals with malaria test results, the majority were: malaria negative (95.3%); female (51.7%); and living on Unguja Island (74.1%). The mean (standard deviation [sd]) age was 21.7 (17.0) years. Out of 21,443 households, 71.9% reported having received IRS in the previous year. Overall, the median weekly rainfall was 7.7 mm (inter quartile range, IQR: 0.0–31.4 mm).

**Table 1:** Annual number of index cases, households followed-up and household members tested by year and Island, 2012–2019

Year	Unguja					Pemba					Total				
	Index cases reported (N)	Index cases investigated: n (%)	Households followed-up	Household members tested (N)	Household members positive: n (%)	Index cases reported (N)	Index cases investigated: n (%)	Households followed-up	Household members tested (N)	Household members positive: n (%)	Index cases reported (N)	Index cases investigated: n (%)	Households followed-up	Household members tested (N)	Household members positive: n (%)
2012^a^	239	97 (40.6)	151	569	35 (6.2)	171	47 (27.5)	48	288	14 (4.9)	410	144 (35.1)	199	857	49 (5.7)
2013	2005	734 (36.6)	1526	8606	503 (5.8)	572	309 (54.0)	475	2,311	148 (6.4)	2577	1043 (40.5)	2001	10,917	651 (5.9)
2014	2815	1449 (51.5)	2571	10,698	592 (5.5)	619	488 (78.8)	604	2973	172 (5.8)	3434	1937 (56.4)	3175	13,671	764 (5.6)
2015	3603	2004 (55.6)	2532	10,205	575 (5.6)	722	616 (85.3)	644	3021	131 (4.3)	4325	2620 (60.6)	3176	13,226	706 (5.3)
2016	2391	1028 (42.9)	1479	5233	263 (5.0)	810	562 (69.4)	766	3,158	81 (2.6)	3201	1590 (49.7)	2245	8391	344 (4.1)
2017	3438	1068 (31.1)	2148	6334	251 (3.9)	700	282 (40.3)	414	1938	73 (3.8)	4138	1350 (32.6)	2562	8272	324 (3.9)
2018	4541	1827 (40.2)	2254	7776	313 (4.0)	1,051	463 (44.1)	713	3280	134 (4.1)	5592	2290 (40.9)	2967	11,056	447 (4.0)
2019	6117	4 310 (70.5)	4237	11,823	411 (3.5)	853	787 (92.3)	881	3808	127 (3.3)	6970	5,097 (73.1)	5118	15,631	538 (3.4)
Total	25,149	12,517 (49.8)	16,898	61,244	2943 (4.8)	5498	3554 (64.6)	4545	20,777	880 (4.2)	30,647	16,271 (53.1)	21,443	82,021	3823 (4.7)

^a^August to December 2012

### Univariate analysis

Factors significantly associated with increased odds of RDT positivity were younger age (*p-value for trend [Ptrend]< 0.001*); self-reported history of fever during the last 2 weeks (odds ratio [OR] = 32.1; 95% CI 29.3–35.1); history of travel outside Zanzibar during the last 30 days (OR = 2.3; 95% CI 2.1–2.4); having more people in the household who travelled outside Zanzibar during the last 30 days (*Ptrend < 0.001*), and living on Unguja Island (OR = 1.2; 95% CI 1.0–1.4). Factors significantly associated with reduced odds of RDT positivity were being male (OR = 0.9; 95% CI 0.8–0.9); sleeping under an LLIN the previous night (OR = 0.7; 95% CI 0.6–0.7); higher household net access (*Ptrend < 0.001*); and living in a household that received IRS in the last 12 months (OR = 0.8; 95% CI 0.7–0.9)) (Table 2).

**Table 2:** Univariate logistic regression analysis of risk factors associated with a positive malaria rapid diagnostic test

Explanatory variable		Total tested	Malaria positive	Malaria positivity (%)	Odds ratio (95% CI)	P-value	P-trend
Individual factors							
Gender (N = 80,532)	Female	41,623	2103	5.1	1.0		
	Male	38,909	1714	4.4	0.9 (0.8–0.9)	< 0.001	
Age group (N = 80,275)	≥ 45 years	9207	181	2.0	1.0		< 0.001
	35–44 years	9016	266	3.0	1.5 (1.2–1.8)	< 0.001	
	25–34 years	11,816	580	4.9	2.5 (2.1–2.9)	< 0.001	
	15–24 years	16,887	838	5.0	2.6 (2.2–3.1)	< 0.001	
	5–14 years	21,948	1311	6.0	3.2 (2.7–3.7)	< 0.001	
	< 5 years	11,401	642	5.6	2.9 (2.5–3.4)	< 0.001	
Self-reported fever in last two weeks (N = 73,273)	No	70,384	2151	3.1	1.0		
	Yes	2889	1354	46.9	32.1 (29.3–35.1)	< 0.001	
History of travel during last 30 days (N = 76,970)	No	55,267	2103	3.8	1.0		< 0.001
	Within Zanzibar	2360	66	2.8	0.8 (0.6–0.9)	0.027	
	Outside Zanzibar	19,343	1506	7.8	2.3 (2.1–2.4)	< 0.001	
Number of household members who travelled outside Zanzibar (N = 82,021)	None	48,966	1751	3.6	1.0		< 0.001
	1	10,977	503	4.6	1.4 (1.3–1.6)	< 0.001	
	≥ 2	22,078	1569	7.1	2.3 (2.1–2.5)	< 0.001	
Slept under an LLIN the previous night (N = 74,119)	No	29,316	1658	5.7	1.0		
	Yes	44,803	1864	4.2	0.7 (0.6–0.7)	< 0.001	
Household factors							
Net access (N = 80,202)^a^	No access	15,756	922	5.9	1.0		< 0.001
	< 80%	24,071	1344	5.6	0.9 (0.8–1.0)	0.058	
	≥ 80%^a^	40,375	1492	3.7	0.6 (0.6–0.7)	< 0.001	
IRS in the last 12 months (N = 82,021)	No	21,934	1173	5.3	1.0		
	Yes	60,087	2650	4.4	0.8 (0.7–0.9)	< 0.001	
Geographical factors							
Zone (N = 82,021)	Pemba	20,777	880	4.2	1.0		
	Unguja	61,244	2943	4.8	1.2 (1.0–1.4)	0.019	
Total weekly rainfall for previous 9 weeks (N = 82,021)	Increments of 10 mm				1.0 (0.9–1.0)	0.757	

CI, confidence interval; LLIN, long-lasting insecticidal net; IRS, indoor residual spraying of households with insecticide

^a^Household level access to LLIN (universal coverage ≥ 80%)

### Multivariate analysis

The VIF was 1.9, suggesting low correlation between the independent variables (explanatory factors). Factors independently associated with increased odds of malaria positivity were younger age (*Ptrend < 0.001*); self-reported history of fever during the last 2 weeks (OR = 35.7; 95% CI 32.3–39.5); history of travel outside Zanzibar during the last 30 days (OR = 2.5; 95% CI 2.3–2.8); and living on Unguja Island (OR = 1.2; 95% CI 1.0–1.5). Factors independently associated with reduced odds of RDT positivity were being male (OR = 0.8; 95% CI 0.7–0.9); having higher household net access (*Ptrend < 0.001*); sleeping under an LLIN the previous night (OR = 0.9; 95% CI 0.7–0.9); and living in a household that received IRS in the last 12 months (OR = 0.8; 95% CI 0.7–0.9). There was significant effect modification when IRS in the last 12 months and sleeping under an LLIN the previous night were combined (OR = 0.7; 95% CI 0.6–0.8) (Table 3).

**Table 3:** Multivariate logistic regression analysis of risk factors associated with a positive malaria rapid diagnostic test (N = 66,756)

Explanatory variable		Odds ratio (95% CI)	P-value	P-trend
Individual factors				
Gender	Female	1.0		
	Male	0.8 (0.7–0.9)	< 0.001	
Age group	≥ 45 years	1.0		< 0.001
	35–44 years	1.4 (1.2–1.8)	0.001	
	25–34 years	2.2 (1.8–2.7)	< 0.001	
	15–24 years	2.4 (1.9–2.9)	< 0.001	
	5–14 years	3.2 (2.7–3.9)	< 0.001	
	< 5 years	2.5 (2.0–3.0)	< 0.001	
Self-reported fever in last two weeks	No	1.0		
	Yes	35.7 (32.3–39.5)	< 0.001	
History of travel during last 30 days	No	1.0		< 0.001
	Within Zanzibar	0.8 (0.6–1.0)	0.104	
	Outside Zanzibar	2.5 (2.3–2.8)	< 0.001	
Malaria prevention				
Net access	No access	1.0		< 0.001
	< 80%	0.8 (0.7–0.9)	0.003	
	≥ 80%*	0.6 (0.5–0.6)	< 0.001	
Slept under an LLIN the previous night	No	1.0		
	Yes	0.9 (0.7–0.9)	0.036	
IRS in the last 12 months	No	1.0		
	Yes	0.8 (0.7–0.9)	0.002	
Interaction term: Slept under an LLIN the previous night and household received IRS in the last 12 months		0.7 (0.6–0.8)	< 0.001	
Geographical factors				
Zone	Pemba	1.0		
	Unguja	1.2 (1.0–1.5)	0.034	

CI, confidence Interval; LLIN, long-lasting insecticidal net; IRS, indoor residual spraying of household with insecticide

## Discussion

This study investigated factors associated with malaria infection (as measured by RDT positivity) among the general population living in households where malaria cases had been detected passively when attending public or private health facilities in Zanzibar. The findings of this study suggest that age, history of fever during the last 2 weeks and history of travel outside Zanzibar during the last 30 days, and living on Unguja Island were independently associated with increased odds of RDT positivity. On the other hand, male gender, sleeping under an LLIN the previous night, higher household net access, living in a household that received IRS in the previous 12 months, as well as combining IRS in the last 12 months and sleeping under an LLIN the previous night were independently associated with reduced odds of RDT positivity.

Age was an important factor within the model, with children aged 5–14 years being at greatest risk of malaria infection. Malaria prevalence in children under 5 years of age has decreased in Tanzania in recent years [15], and other studies in mainland Tanzania suggest the risk of malaria infection is now higher in school-aged children compared to children under 5 years of age [6, 16]. The finding of this study might partially be explained by the association between age and those who slept under an LLIN the previous night, with a lower proportion of children aged 5–14 years (55.2%) and young adults aged 15–24 years (47.5%) having been observed to sleep under LLINs, compared to children < 5 years [6, 9, 17]. Given their higher odds of having a positive RDT result, targeting interventions to improve LLIN access and use in children aged 5–14 years and young adults aged 15–24 years will thus be important in Zanzibar, since these age groups appear to be at greater risk of infection and are likely to contribute to on-going malaria transmission. Our analysis also showed that males had reduced odds of having a positive RDT result. Typically, an increased, gender-associated risk for a vector-borne disease such as malaria suggests gender-related differences in behaviour (e.g., night time work or leisure activities) [18]. It is likely that the reactive case detection through HSaT was systematically missing males with increased risk of malaria because only residents present at time of DMSO visit are tested., Nonetheless, further studies are needed to investigate specific factors that could explain the higher odds of malaria RDT positivity among females observed in our study.

As reported previously [8, 16, 19, 20], history of fever during the last 2 weeks was significantly associated with having a positive RDT result. While self-reported fever as a proxy for symptomatic malaria infection is prone to recall and/or reporting bias and thus lacks both the sensitivity and specificity, particularly in elimination settings [20, 21], the finding suggests there is a subset of the population who do not present to health facilities when they experience malaria symptoms such as fever. Therefore, persistent messaging on recognizing malaria symptoms and prompting care-seeking behaviour at health facilities is needed even in a low transmission setting such as Zanzibar. The finding also reinforces the continued need for timely RCD, including the testing of all household members in index case households and possibly neighbouring households with or without malaria symptoms.

The results of this study confirm prior studies showing that individuals who travelled outside Zanzibar during the last 30 days had increased odds of a positive RDT result at the time of the household investigations [10]. Malaria infection associated with travel (“malaria importation”) is frequently considered a concern in low transmission or elimination settings [22–25]. There is evidence that the majority of people travelling outside Zanzibar head to malaria endemic areas on the Tanzania mainland [23]. Therefore, implementing control measures to prevent infection among Zanzibaris visiting the mainland, as well as timely detection of travel-associated malaria cases and controlling onward transmission will be key to sustaining the low malaria burden and progressing towards malaria elimination in Zanzibar. Several approaches to limit the size and impact of travel-associated malaria could be considered, including targeting information and education messaging to potential travellers regarding the risks of malaria when travelling, malaria symptomatology, and the possible approaches for individual protection. Advice for travellers to malaria endemic locations could include recommendations to sleep under an LLIN every night, use mosquito repellents, and possibly take anti-malarial chemoprophylaxis depending on the travel destination and malaria risk. An approach to early detection for travel-associated malaria might involve screening every person arriving at key border entry points using ultra-sensitive diagnostic tests such loop-mediated isothermal amplification polymerase chain reaction, and when clinically appropriate treating or referring positive individuals. Such approach could also reinforce malaria surveillance and education on malaria prevention among travellers at these entry points.

The reduced odds of RDT positivity independently associated with LLIN access and use shown here adds to the vast body of evidence supporting the effectiveness of LLIN in their protection against malaria and other vector-borne diseases [26]. This study also found that increased availability of LLINs in households was associated with a reduced odd of RDT positivity, with universal coverage (net access ≥ 80%) having the greatest protective effect [27]. Nonetheless, having access to LLINs at household level is not sufficient, and approaches to encourage at-risk populations to consistently sleep under an LLIN, even in a low transmission setting like Zanzibar, are essential.

The protective effect of IRS reported in this study was within the range reported by other studies [19, 28, 29]. The study findings suggest that IRS reduces the risk of malaria infection in the household; however, IRS operations are expensive and require considerable human resources. IRS operations in Zanzibar started in 2006, with different classes of insecticides used over the past 14 years [30]. To mitigate programmatic costs and limit the expansion of insecticide resistance, Zanzibar has shifted from a blanket IRS approach used between 2006 and 2012 to a more targeted and focal approach from 2015 onwards, whereby only areas experiencing a high number of cases and malaria incidence are prioritized.

Combining IRS and LLINs has increasingly become a common approach to control malaria in sub-Saharan Africa. This study provides further evidence of the added benefit offered to individuals when combining IRS and LLINs [31, 32]. While people who lived in households where IRS was applied in the last 12 months were less likely to be RDT positive compared to those who were not living in households where IRS has been applied, regardless of whether they slept under a LLIN or not, they were even less likely to test RDT positive if sleeping under an LLIN. This suggests that even in low transmission settings, the possibility of combining these two vector control interventions is effective.

This study has a number of potential limitations. Firstly, the analysis included members living in the same households as passively detected malaria cases (index cases); therefore, it is expected that this population may be at greater risk of malaria compared to the general population in Zanzibar. Secondly, only two thirds of the 30,647 passively detected cases were followed-up for HSaT; therefore, this may be a source of bias since most of the index cases lost-to-follow-up were detected during the peak malaria transmission season. Thirdly, RDTs’ have a limited sensitivity and are likely to miss a large proportion of infections, since in Zanzibar’s elimination setting most of these are sub-clinical, low-parasite density infections [21]. The RDTs’ low sensitivity during RCD is a potential source of bias because they are designed to identify symptomatic malaria cases that are typically high-density infections. This low RDT sensitivity has broader programmatic implications, with Zanzibar possibly having to consider the use of more sensitive diagnostics (e.g., Polymerase chain reaction [PCR]), in addition to other strategies such as focal mass drug administration or seasonal malaria chemoprevention, to eliminate malaria [3]. Finally, a fifth of the individuals with malaria test results were missing one or more explanatory variable; however, sensitivity analysis showed that this missingness was not a source of bias.

## Conclusions

This study has identified risk factors associated with malaria infection (as measured by RDT positivity) that could be addressed through increased surveillance and targeted interventions. This study found that between 2012 and 2019 children aged 5–14 years, females, residents of Unguja and residents who travel outside of Zanzibar had greater odds of having a positive RDT result.

These findings suggest that even in a low transmission setting like Zanzibar, vector control remains an important malaria prevention intervention and underscores the need to maintain universal access to LLIN, persistent promotion of LLIN use, and IRS. In addition to continued effective coverage and use of vector control interventions, enhanced behavioural change and preventive strategies specifically targeting children aged 5–14 years and travellers could enhance Zanzibar’s progress towards malaria elimination.

## Data Availability

The datasets used and /or analyzed during the current study are available from the corresponding author on reasonable request.

## Abbreviations

ACT: Artemisinin-based combination therapy
CI: Confidence interval
CMSO: Council malaria surveillance officer
HSaT: Household-based screening and treatment
IRS: Indoor residual spraying
IQR: Inter quartile range
LLIN: Long-lasting insecticidal net
MCN: Malaria case notification (system)
RDT: Rapid diagnostic test
OR: Odds ratio
PCR: Polymerase chain reaction
RCD: Reactive case detection
VIF: Variance inflation factor
ZAHRI: Zanzibar Health Research Institute
